# A novel therapeutic vaccine targeting the soluble TNFα receptor II to limit the progression of cardiovascular disease: AtheroVax™

**DOI:** 10.3389/fcvm.2023.1206541

**Published:** 2023-07-18

**Authors:** Patrick L. Iversen, Nicholas Kipshidze, Nodar Kipshidze, George Dangas, Eduardo Ramacciotti, Zurab Kakabadze, Jawed Fareed

**Affiliations:** ^1^ Department of Environmental and Molecular Toxicology, Oregon State University, Corvallis, OR, United States; ^2^ New York Cardiovascular Research, New York, NY, United States; ^3^ Mailman School of Public Health, Columbia University, New York, NY, United States; ^4^ Department of Cardiology, Icahn School of Medicine at Mount Sinai, New York, NY, United States; ^5^ Science Valley Research Institute, Sao Paulo, Brazil; ^6^ Head Department of Clinical Anatomy, Tbilisi State Medical University, Tbilisi, Georgia; ^7^ Department of Molecular Pharmacology and Neuroscience, Loyola University Medical Center, Maywood, IL, United States

**Keywords:** atherosclerotic cardiovascular disease, tumor necrosis factor alpha, TNFRII, soluble TNFRII, therapeutic vaccine, peptide vaccine

## Abstract

The burden of atherosclerotic cardiovascular disease contributes to a large proportion of morbidity and mortality, globally. Vaccination against atherosclerosis has been proposed for over 20 years targeting different mediators of atherothrombosis; however, these have not been adequately evaluated in human clinical trials to assess safety and efficacy. Inflammation is a driver of atherosclerosis, but inflammatory mediators are essential components of the immune response. Only pathogenic forms of sTNFR2 are acted upon while preserving the membrane-bound (wild-type) TNFR2 contributions to a non-pathogenic immune response. We hypothesize that the inhibition of sTNRF2 will be more specific and offer long-term treatment options. Here we describe pre-clinical findings of an sTNFR2-targeting peptide vaccine (AtheroVax™) in a mouse model. The multiple pathways to synthesis of the soluble TNFRII receptor (sTNFRII) were identified as sTNFRII(PC), sTNFRII(Δ7), and sTNFRII(Δ7,9). The sTNFRII(Δ7) peptide, NH2-DFALPVEKPLCLQR-COOH is specific to sTNFR2 based on an mRNA splice-variant in which exon 6 is joined to exon 8. The role of sTNFRII(Δ7) as a mediator of prolonged TNFα activity by preventing degradation and clearance was investigated. Inflammation is a critical driver of onset, progression and expansion of atherosclerosis. The TNFα ligand represents a driver of inflammation that is mediated by a splice variant of TNFR2, referred to as sTNFRII(Δ7). The multiple forms of TNFRII, both membrane bound and soluble, are associated with distinctly different phenotypes. sTNFRII(PC) and sTNFRII(Δ7) are not equivalent to etanercept because they lack a clearance mechanism. The unique peptide associated with sTNFRII(Δ7) contains a linear B-cell epitope with amino acids from both exon 6 and exon 8 supporting the vaccine design. Animal studies to evaluate the vaccine are ongoing, and results will be forthcoming. We describe a peptide vaccine targeting sTNFR2 in limiting the progression of atherosclerosis. A therapeutic vaccine limiting the progression of atherosclerosis will greatly contribute to the reduction in morbidity and mortality from cardiovascular disease. It is likely the vaccine will be used in combination with the current standards of care and lifestyle modifications.

## 1. Introduction

A therapeutic atherosclerosis vaccine with the objective to prevent or reduce development and progression of atherosclerosis has the potential to reduce incidence of heart attack and stroke. Atherosclerosis is characterized by lipid-rich plaques in large and medium-sized arteries that appear to originate from a chronic inflammatory response. The progressive plaque growth, plaque rupture or erosion with subsequent thrombus formation can lead to arterial occlusion and cardiovascular disease (CVD). The approach to a therapeutic atherosclerosis vaccine is to target are reduce levels of molecules that contribute to the development and progression of atherosclerosis.

Current measures to prevent and treat CVD include cholesterol-lowering, antiarrhythmic, and antihypertensive drugs which coupled with bypass surgery and percutaneous interventions have significantly reduced CVD mortality. Emerging vaccines offer a new dimension in the treatment of CVD. Promising preclinical studies with vaccines targeting PCSK9 (1) and oxidized LDL (2) reveal reduced atherosclerotic plaque formation in animal models. The results support the need for broader research and evaluation of vaccine safety and efficacy in humans. Finally, even if a therapeutic atherosclerosis vaccine is developed, it will likely be used in combination with the current standards of care.

Patients with myocardial infarction or high-risk heart disease that received influenza vaccine within 72 h of an invasive coronary procedure had a lower risk of all-cause death, MI, or stent thrombosis, and cardiovascular death 1 year after vaccination (3). The influenza vaccine is associated with lowering proinflammatory cytokines (4) and may exert anti-inflammatory and plaque stabilizing effects (5). The association of influenza vaccine with CVD supports the role of chronic inflammation as a central driver of CVD. Control of chronic inflammatory mediators is likely to be key to reducing CVD (6).

Cardiac inflammatory signaling is complex and multifaceted including triggers from viral infections and biochemical stresses. Numerous anti-inflammatory agents are available but have so far had limited success in the treatment of CVD (Table 1).

**Table 1:** Current anti-inflammatory therapy for atherosclerotic cardiovascular disease.

Class	Examples	Comments
Cyclooxygenase (COX) inhibitors	Aspirin, ibuprofen, naproxen	Other than aspirin may increase risk of heart attack and stroke
Arachidonate 5-llipoxygenase	Licofelone	
Immune selective (ImSAIDs)		
TNFα inhibitors	Remicade and Humera	Arthritis; Antidrug antibodies limit use
TNFα receptor Fc fusion	Etanercept (Enbrel)	Used in psoriasis
IL-6 MAb	Toculizumab	Used in Juvenile arthritis and COVID-19
IL-12/23 MAb	Ustekinumab	Used in Crohn's and psoriasis
IL-1/IL-1 receptor	Anakinra/Rilonacept	Autoimmune disease
Immunosuppressants	Glucocorticoids Calcineurin Inhibitors- tacrolimus mTOR inhibitors- sirolimus Methotrexate	Short term regimens
Immune signal transduction modulators	Tyrosine kinase inhibitors-Gleevec Janus kinase inh.- Barcitinib PPAR agonists- troglitazone	
Anti-gout agents	Colchicine	Acute treatment regimens

## 2. Tumor necrosis factor-α (TNF-α)

The tumor necrosis factor (TNF) super-family is composed of 19 different ligands and 29 receptors. This super-family is a pivotal component in cellular signaling cellular differentiation, survival and death (7). Immune responses are downstream to TNF signals involving both innate and adaptive immune cells. The interplay of ligand and receptors is complex and when dysregulated, inflammatory and autoimmune consequences can result.

Tumor necrosis factor-α (TNF-α) expression is triggered by the immune system response to pathogens and their associated toxins. TNFα is synthesized as a transmembrane precursor protein (mTNF-α) as a 26 kDa (233 amino acids, a 76 amino acid leader and 157 amino acid body). The mTNF-α is then proteolytically cleaved by the TNF-α converting enzyme (TACE or ADAM17) to create a free soluble homotrimer sTNF-α as 17 kDa monomers. TNF-α interacts with two receptors: TNFR1 known as CD120a and p55 and TNFR2 known as CD120b and p75. These receptors also bind lymphotoxin alpha (LT-α). The extracellular domain of TNFR2 is composed of 4 cysteine rich domains (CRD) identified as CRD1, CRD2, CRD3, and CRD4. TNFR2 is in a trimeric form as it sits on the cell membrane. Binding TNF-α activates the TNFR2 cytoplasmic domain. This TNFR2' signals through TRAF2 and induces JNK and NF-kB activation. TNFR1 may mediate bacterial and viral challenges but TNFR2 is primarily involved in response to viral challenges.

TNF-α is one of the most potent pro-inflammatory cytokines. Biosynthesis is controlled at a post-transcriptional stage through the competitive binding of tristetraprolin (TTP) to an AU-rich untranslated region in the 3'-region of the mRNA. Dephosphorylated TTP binds the mRNA and promotes degradation and phosphorylation weakens TTP affinity for mRNA. Stimuli such as lipopolysaccharide (LPS), interleukin 1β (IL-1β), IL-6, interferon gamma (IFN-γ), tissue trauma or hypoxia regulate translocation of TTP from the nucleus to the cytoplasm resulting in enhanced TNF-α biosynthesis. Mitogen-activated protein kinases (MAPK) is involved in control of numerous genes including nuclear factor kappa B (NF-κB) which positively regulates the promoter of TTP.

TNF-α promotes insulin resistance leading to obesity and type 2 diabetes. It is used by the immune system as a signal released by macrophages to coordinate an inflammatory response. However, TNF-α is also produced by mast cells, endothelial cells, cardiac myocytes, adipose tissue, fibroblasts, and neurons. Resulting immune responses include fever, apoptotic cell death, cachexia (wasting syndrome), and inflammation (heat, swelling, redness, and pain) while inhibiting tumorigenesis and infections.

### 2.1. TNF-α agonist

Initially, a cytotoxic factor produced by lymphocytes, lymphotoxin, was reported by two independent groups in 1964 (8). The name tumor necrosis factor was reported for a cytotoxic factor produced by macrophages in 1974. These cytotoxic factors were identified based on their ability to kill mouse fibrosarcoma cells (9). Excessive production of TNF was associated with the cause of malaria disease and endotoxin poisoning (10). Subsequent studies have identified production in cell types beyond the macrophage to include lymphoid cells, mast cells, endothelial cells, cardiac myocytes, adipose tissue, fibroblasts, and neurons.

TNF-α is used as an immunostimulant drug, Tasonermin, in the treatment of certain cancers with modest effectiveness. The soluble sTNF-α is also administered therapeutically from recombinant (rhTNF-α; amino acids 77-233) production methods. The inhibition of PBMCs by rhTNF-α, IC_50_, is 1,728 ± 11 ng/ml and the effective concentration, EC_50_, is 250 ± 1 ng/ml. The IC_50_ in Caco-2 cells was 297 ng/ml, HepG-2 cells was 197 ng/ml, and MCF-7 cells was 374 ng/ml (11).

TNF-α mRNA half-life was 75.3 ± 16.7 min (Table 2) in keratinocytes treated with UVB alone and 56.0 ± 4.5 min following UVB plus IL-1-α (not significantly different). No TNF-α mRNA was detected in keratinocytes following sham treatment (12). The half-life of TNF-α mRNA is 140 min in primary alveolar macrophages in control media. The mRNA is stabilized by cigarette smoke extract, the half-life is prolonged to well over 300 min (13). An immediate-early response gene, tristetraprolin (TTP), can destabilize the TNF-α mRNA.

**Table 2:** TNFα half-life observations.

Measure	Half-life (hours)	Comments
TNFα mRNA	0.9–2.3	Can be reduced post transcriptionally by tristetraprolin (TTP)
sTNFα protein	1.2–2.1	Clearance by multiple mechanisms

Administration of 2 µg/kg/0.5 h in rats resulted in a plasma half-life of 5–8 min. Administration of doses between 10 and 500 µg/kg/0.5 h by intravenous infusion in rats resulted in dose independent plasma half-life of about 0.5 h (14). The half-life for rhTNF-α in rhesus macaques was between 1.2 and 2.1 h with doses of 10, 20, 30, and 120 µg/kg/0.5 h. The NHP studies suggested two different elimination mechanisms for TNF-α clearance, a nonspecific non-saturable process, and a specific saturable process (15).

The sTNF binds TNFR1 with Kd = 1.9 × 10^−11^ M but TNFR2 with Kd = 4.2 × 10^−10^ M and the sTNF/TNFR1 complex depends on stabilization while the sTNF/TNFR2 complexes are short lived and may not be signaling competent (16). TNFR2 signaling is best activated by mTNF/TNFR2 complexes.

TNF-α appears to have contradictory effects on almost every type of cancer. Pro-tumorigenic effects include proliferation, promotion through TNFR1 and IL-17, immunosuppression, progression cell survival from reverse signaling, and metastasis through p38 MAPK, Erk1/2 and β-catenin. Anti-tumorigenic effects include tumor cell apoptosis, inhibition of proliferation, and generation of specific cytotoxic CD8 + lymphocytes (17).

### 2.2. TNF receptors

Binding TNFR1 leads to assembly of a complex of TNF receptor 1 associated protein with death domain (TRADD), the receptor associated factor 2 (TRAF2), the receptor interacting protein kinase (RIP1), and the cellular inhibitor of apoptosis proteins (cIAPs) 1 and 2. The selection of intracellular binding partners is influenced by the cIAP in the complex. Adapter signaling can produce several different signals: (1) a death inducing signaling complex (DISC) composed of TRADD/fas associated death domain (FADD)/procaspase 8 complex resulting in apoptosis, (2) phosphorylation of IKKβ leading to nuclear localization of nuclear factor kappa B (NFκB) free dimers and transcriptional activation of the NFκB pathway, and (3) recruitment of the necrosome complex leading to membrane permeabilization and necroptosis.

The TNFR2 signaling complex lacks the death domain but does include cIAP1/cIAP2 and signal through non-canonical activation of NFκB, the c-jun N-terminal kinase (JNK), p38 MAPK, and lipid phosphatidylinositol-4,5-bisphosphonate (PIP2) to produce proliferation of regulatory T cells (Treg) and activation of PKB/Akt in promoting cell survival and proliferation. Broadly, the actions of TNFR1 and TNFR2 have opposing effects on the immune system.

### 2.3. TNF-α inhibitors

TNF-α inhibitors include a spectrum of monoclonal antibodies: (a) Infliximab (Remicade) is a mouse Fv domain fused to a human Fcγ1 IgG1, (b) adalimumab and golimumab are human Fv domains with human Fcγ1 IgG1, (c) etanercept (Enbrel) is the four cysteine-rich domains (CRD) domains of TNFR2 fused to human Fcγ1 IgG1, and (d) certolizumab pegol is a humanized Fv (from murine) Fab' with the hinge region cross-linked to two 20 kDa molecules of polyethylene glycol. The current indications for anti-TNF-α include immune-mediated inflammatory diseases (IMID) such as rheumatoid arthritis, Crohn's disease, juvenile idiopathic arthritis, plaque psoriasis, ankylosing spondylitis, ulcerative colitis, and non-infectious uveitis (Table 3).

**Table 3:** Comparison of approved TNF-α inhibitors.

Agent	Half-life (D)	ADA (%)	Nab (%)	Regimen	Cost/QALY
Remicade (infliximab)	9.0–20	51.2	85.6	IV Q 8 weeks	$79,518/12.34 (18)
Humera (adalimumab)	9.6	93.4	41.4	SQ Q- 2 weeks	91,695/13.25
Enbrel (etanercept)	2.4–3.9	0–7	0	SQ 2x/week	$87,441/11.79
Cimzia (vertolizumab)	14	51.2	>60	SQ/IV Q-2 weeks	

Remicade (infliximab) a chimeric monoclonal antibody binding TNF-α is administered in combination with methotrexate (MTX 10–25 mg/wk PO) at a dose of 3–5 mg/kg IV every 8 weeks. Important limitations include antidrug antibodies (ADA) which are observed in 51.2 percent of patients in a 30-week treatment period and neutralizing antibodies (Nab) are observed in 85.6 percent of patients in a 30-week treatment period (19). A meta-analysis of the half-life of Remicade was observed to range from 9.0 to 20.3 days but clearance is enhanced by 48 percent in patients with ankylosing spondylitis compared to rheumatoid arthritis. Clearance is also enhanced in individuals with ADA (20).

Humera (adalimumab) is a recombinant human monoclonal antibody that binds TNF-α administered as a 40 mg dose SQ. The elimination half-life was 231.9 h in adult male healthy volunteers with a 71 percent coefficient of variation (21). This study identified 93.4 percent of patients with ADA by day 70 and 41.4 percent of patients with Nabs by day 70.

Etanercept is a 75 kDa fusion protein capable of binding TNF-α 50- to 100-fold more potent than the endogenous TNFR1/R2. Enbrel was evaluated in clinical trials beginning in 1993 with approval for RA in 1998. A human dose of Enbrel (Etanercept) is 50 mg in 1 ml administered subcutaneously once a week. The dose results in a Cmax of 3,151 ± 1,261 ng/ml and a half-life 94.6 ± 19.2 h in healthy adults (22). A dose ranging study in patients with rheumatoid arthritis (RA) observed an elimination half-life of 57.8 ± 26.1 h at 50 mg SQ twice-weekly to 68.2 ± 27.4 h at 10 mg SQ once weekly. The observed EC_50_ was between 465 and 573 ng/ml and a steady state concentration of 1,170 ng/ml was observed in patients receiving 50 mg once-weekly (23). A 3 year follow-up study reveals 4.1 percent of patients fail to adhere to etanercept therapy due to medical reasons (24). Patients treated with Enbrel are at increased risk for developing serious infections leading to hospitalization and death.

Cimzia (Certolizumab Pegol) is a humanized monoclonal antibody conjugated to polyethylene glycol Fab fragment. The dose of 200 mg every other week (up to 800 mg) SQ and 10 mg/kg IV are administered with an elimination half-life of approximately 14 days and clearance is enhanced in patients with inflammatory conditions such as RA (25). Incidence of ADA was about 65 percent and over 97 percent of the response led to Nabs (26).

Some patients taking TNF-α inhibitors develop aggravation of disease and new onset of autoimmunity. This contradictory action suggests an immunosuppressive action of TNF-α which some ascribe to enhancement of regulatory T-cells (Tregs) due to binding TNFR2. One of the key limitations with inhibitors is the increased risk of infection such as tuberculosis (TB), development of autoimmune diseases and lymphomas (27). In addition, current inhibitor therapies often induce production of ADAs and specific NAbs leading to diminish inhibitor efficacy.

## 3. TNF-α and atherosclerosis

Animal models provide insights into the association between TNF-α and heart specific inflammation. Mice fed an atherogenic diet are protected from atherosclerotic lesion formation in *Tnfa*^−/−^ mice and exclusive expression of mTNF-α reduces the inflammatory response (28). *Apoe*^−/−^ mice fed an atherogenic diet show reduced plaque growth in *Tnfa*^−/−^ mice (29). In the ischemia reperfusion model, mice show lower infarct area and improved cardiac functions in *Tnfa*^−/−^ mice (30). Mice infected with coxsackievirus B3 to induce myocarditis have reduced myocarditis but no changes in virus titer in *Tnfa*^−/−^ mice (31). The animal model data reveal a dual role of TNF-α and opposing effects of TNFR1 and TNFR2. These experimental data address the clinical failure of TNF-α inhibitors in heart failure patients and further suggest targeting TNFR2 over TNF-α.

TNFR1 and TNFR2 have opposing effects in the heart. TNFR1 exacerbates hypertrophy, inflammation, and cell death in heart failure but TNFR2 limits these events (32). TNFR1 aggravates ventricular remodeling but TNFR2 improves the action after myocardial infarction.

TNF-α is associated with increased risk of coronary heart disease development (33). IMID patients have increased risk of atherosclerotic cardiovascular events. Chronic inflammation leads to acceleration of atherosclerosis (34). Myocardial infarction rates are reduced in rheumatoid arthritis patients taking anti-TNF-α therapies (35, 36). However, enthusiasm for use of TNF-α inhibitors for treatment of patients with cardiovascular risk remains controversial as a few large multi-center comparative studies fail to demonstrate benefit for cardiovascular-related events, heart failure, death risk and improved cardiovascular outcomes (37–39).

The apparent disconnect between animal models of coronary heart disease and outcomes from human studies using TNF-α inhibitors may be due to limited target specificity of TNF-α inhibitors. However, use of TNF-α inhibitors is associated with multiple limitations.

## 4. Soluble TNFR2

**Figure 1:**
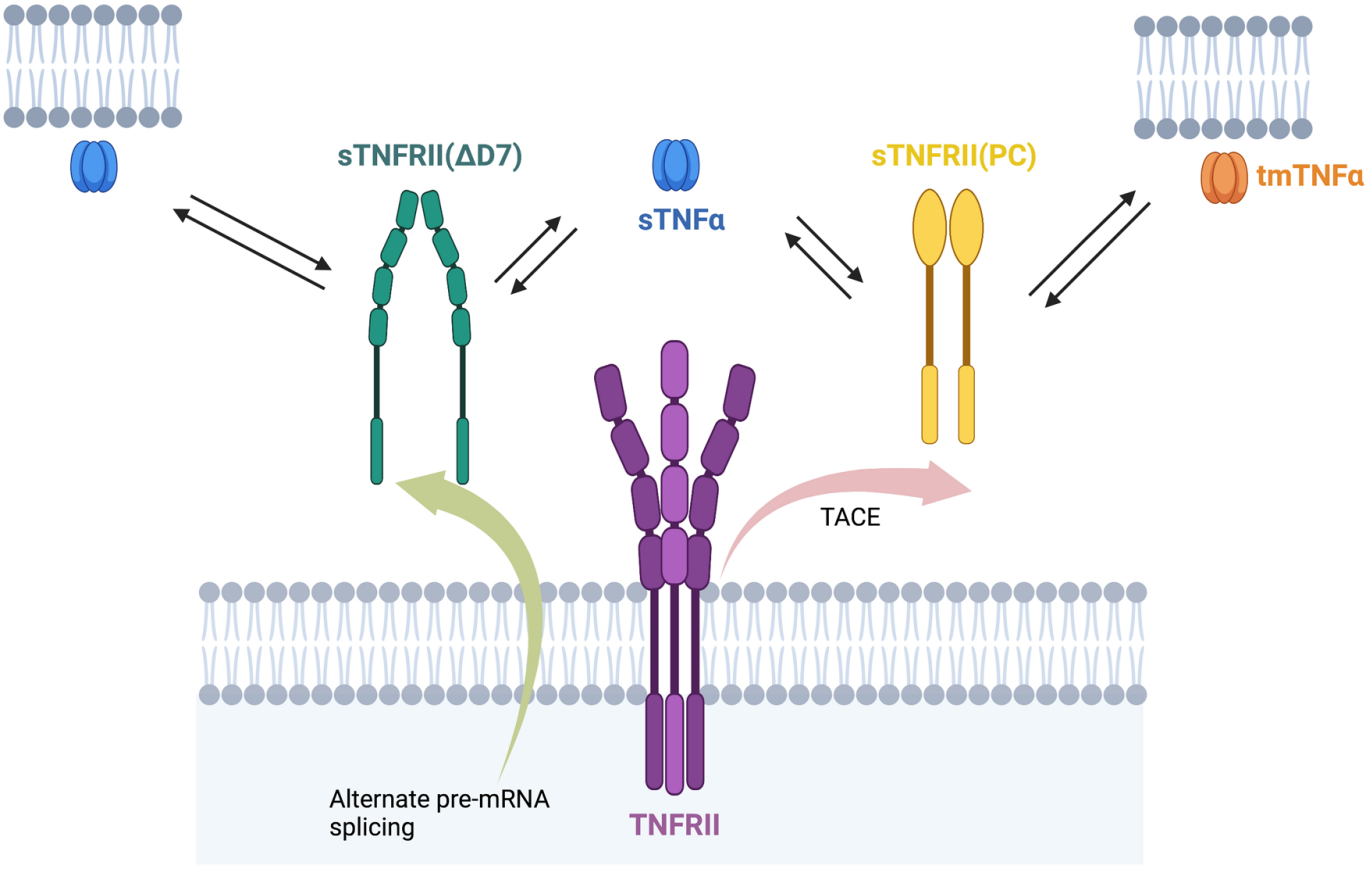
sTNFRII(PC), sTNFRII (Δ7), and sTNFRII(Δ7,8) bind sTNFα and tmTNFα. The multiple forms of the TNFRII, both membrane bound and soluble, can all bind either a membrane bound or soluble form of TNFα. This diversity of binding proteins leads to complexity in TNFα stability and signaling.

Levels of sTNFRs are associated with increased mortality and morbidity in a diverse range of human diseases (Figure 1). The membrane bound TNFRs may be derived by proteolytic cleavage by TNF-α converting enzyme (TACE). They retain ligand binding activity as soluble receptors. T676G SNP (M196R) in exon 6 of the TNFR2 gene is associated with enhanced sTNFRs released by T cells in RA. The SNP was not associated with radiographic or functional severity of RA. This variant shows significantly lower induction of NF-κB and enhanced TNFR1 signaling of apoptosis (40).

sTNFRs are associated with sites of inflammation such as arthritis in Bechet's disease (41). sTNFR2 is a marker of cardiovascular disease in people with diabetes (42). sTNFR2 levels were associated with DNA methylation (epigenetic regulation) in circulating lymphocytes from participants in the Framingham Heart Study (43). A study of 48 patients with ST-elevation myocardial infarction (STEMI). In STEMI patients, circulating levels of sTNFR1 and sTNFR2 are associated with infarct size and LV dysfunction. It appears they play a role in apoptosis in ischemia-reperfusion injury (44). A study of 131 patients with chronic kidney disease (CKD) were evaluated. They conclude sTNFR1 (HR 1.51) and sTNFR2 (HR 1.13) are independently associated to all-cause mortality or an increased risk for cardiovascular events in advanced CKD irrespective of the cause of kidney disease (45). Increased concentrations of circulating TNFR1 and TNFR2 (sTNFR1 and sTNFR2) were associated with increased risks of cardiovascular events and mortality in patients with stable coronary heart disease (46).

Psoriasis patients treated with TNFα inhibitors produce more sTNFα and sTNFR2 and patients not responding produce higher levels of both sTNFα and sTNFR2 (47).

**Figure 2:**
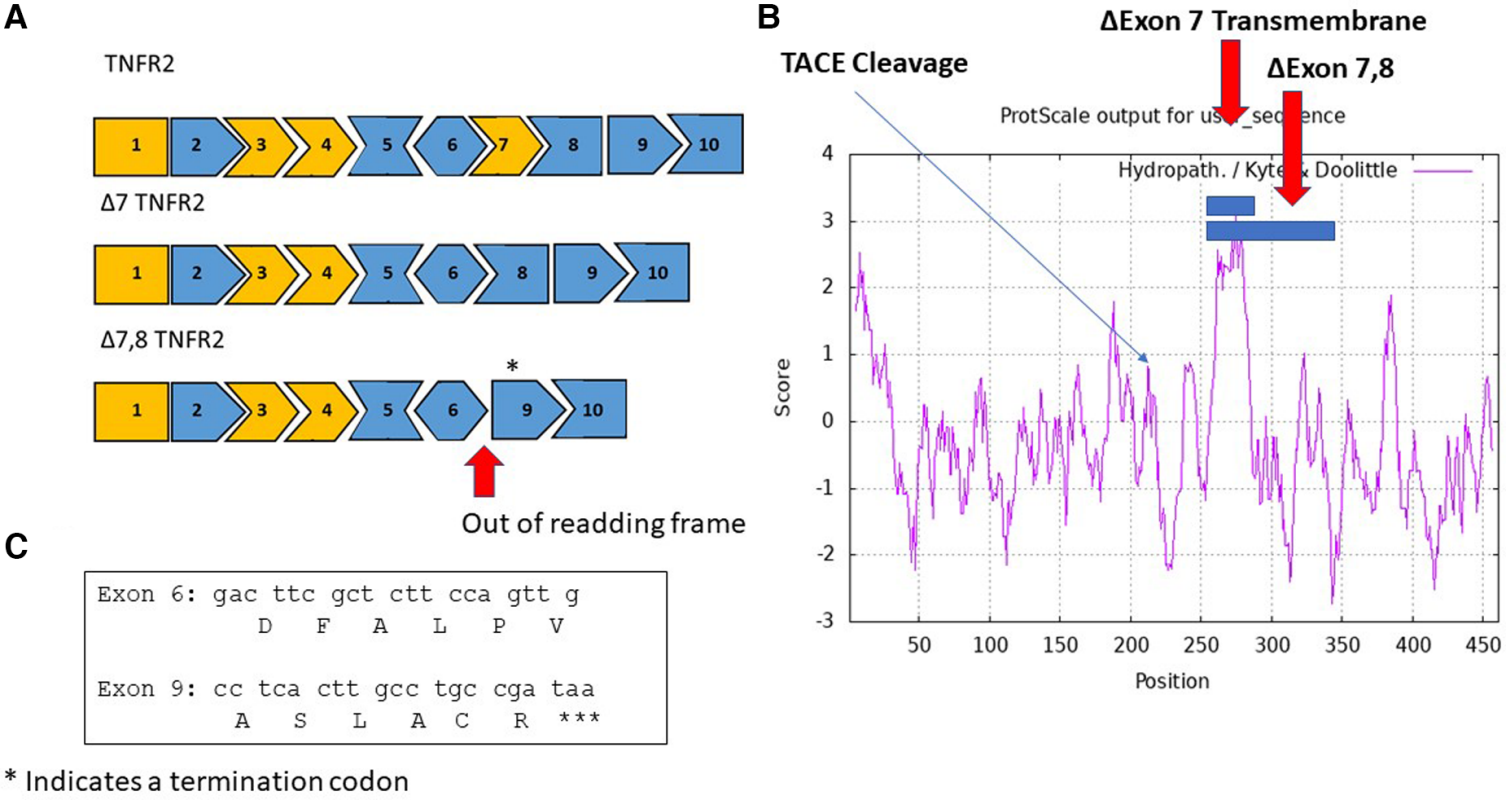
Alternate splice variants of TNFR2 include sTNFRII (Δ7), and sTNFRII(Δ7,8). (**A**) An exon map of the TNFRII displays the 10 exons in the gene with cassette exons indicated in orange. Two splice variants are shown, an in-frame variant in which exon 7 is skipped resulting in the sTNFRII(Δ7) variant and an out of frame variant sTNFRII(Δ7,8). The asterisk above exon 9 indicates the location of a termination codon TAA. (**B**) A hydropathy map of TNFRII with blue bars indicating amino acids exclude associated with exons 7 and 7 plus 8 are located in the transmembrane domain. An arrow indicates the location of the TACE cleavage site responsible for releasing the extracellular domain of sTNFRII(PC). (**C**) The nucleic acid and amino acid sequences joined in the sTNFRII(Δ7,8) splice variant.

A soluble TNFR2 is also synthesized by alternate splicing of pre-mRNA (Figure 2) in response to inflammation (48). The splice variant lacks exon 7 and 8 that encode the transmembrane domain of TNFR2. An antisense strategy has been evaluated to induce skipping of exon 7 of TNFR2. The exon skipping strategy demonstrated amelioration of symptoms in a collagen-induced arthritis mouse model and promoted survival in a TNF-α induced hepatitis mouse model (49). A few key limitations with the antisense study include the oligonucleotide signaling through Tol-receptors leading to NFκB signaling, inability to discriminate between TACE and antisense induced sTNFR2, and limited quantities of Δ7-TNFR2 protein produced. While therapeutically challenging, an antisense oligonucleotide can be utilized as a control to produce the target sTNFR2 antigen in animal models.

### 4.1. sTNFRII(Δ7) role in inflammation

TNF-α is a central regulator of the inflammatory response. Therapeutic interventions include both agonists and antagonists. As an agonist, both the mRNA encoding TNF-α and the translated protein have short half-lives so chronic effects such as rheumatoid arthritis or atherosclerosis require chronic gene expression. TNF-α can produce contradictory and opposite effects in multiple circumstances. The diversity of TNF-α effects appears to be associated with the two receptors, TNFR1 and TNFR2 with therapeutic intervention focus on TNFR2.

**Figure 3:**
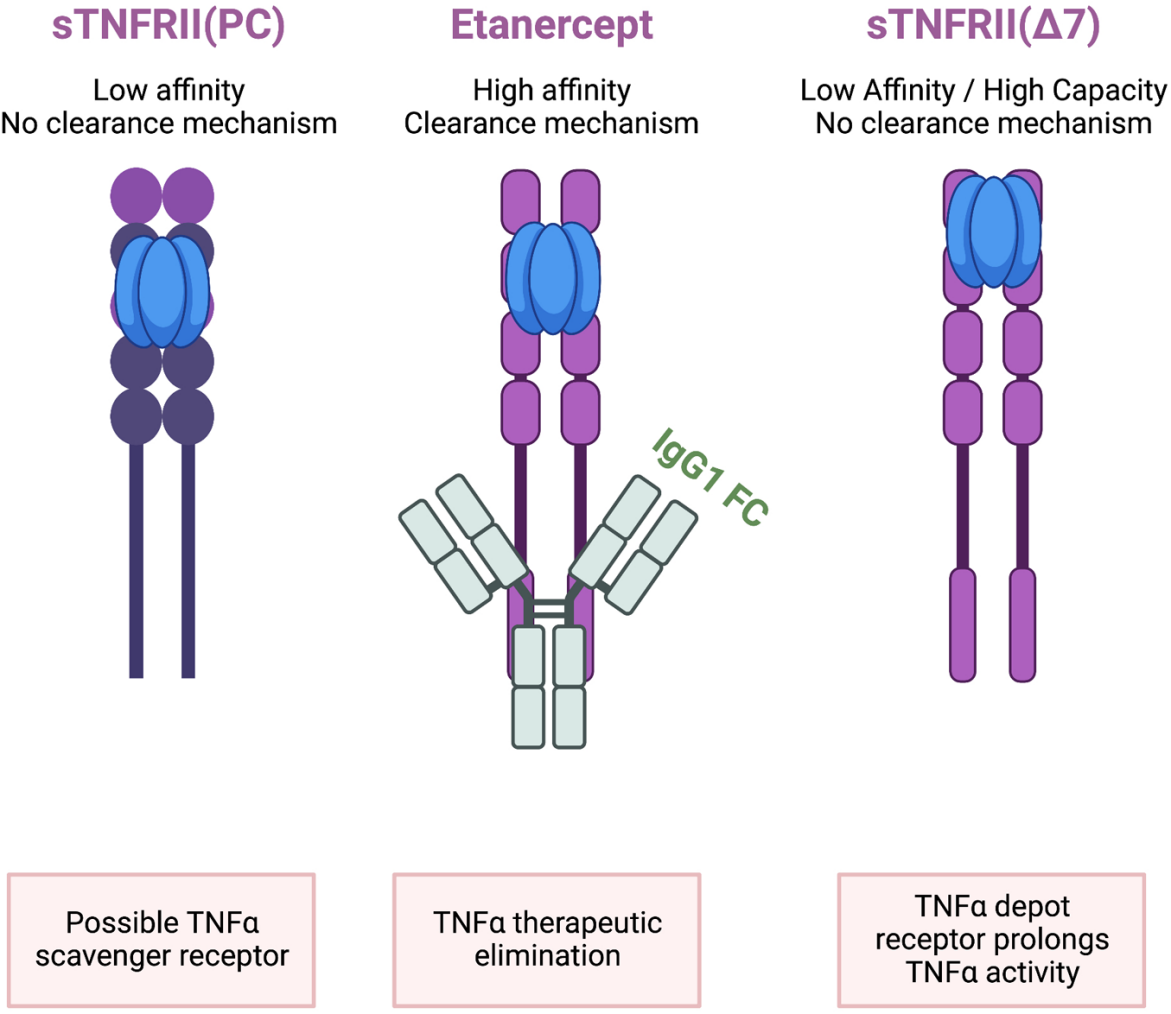
sTNFRII (Δ7) is not equivalent to etanercept or sTNFRII(PC). All three molecules contain the four extracellular CRD regions and can bind TNFα. Only Etanercept has a clearance mechanism provided by the fusion with a human IgG1 Fc region capable of removing bound TNFα from circulation. The relative abundance of sTNFRII(Δ7) is on the order of 2,500 ng/ml which is in excess of TNFα in healthy individuals which is on the order of 0.08 pg/ml. The low affinity but high-capacity binding is proposed to bind TNFα and protect it from rapid degradation based on the 1–2 h half-live of TNFα. The net effect is that the sTNFRII(Δ7) is a depot reservoir for TNFα extending the proinflammatory activity.

The association of TNFR2 with pathogenesis in humans and animal models is complicated by limitations in measures of the soluble TNFR2 in the context of membrane TNFR2. The appearance of a soluble TNFR2 in blood may lead to two opposing interpretations (Figure 3). First, the most frequently reported action is to bind TNFα as a “decoy” receptor, an endogenous TNFα inhibitor, resulting in lower free sTNFα levels and reduced inflammation. The opposing interpretation also involves binding free sTNFα but the outcome is stabilization of sTNFα by protecting it from clearance and degradation leading to prolonged inflammation. The second interpretation proposes the free sTNFα plus sTNFR2 bound sTNFα are physiologically relevant. Reference TNFα levels in healthy individuals were 0.083 ± 0.14 pg/ml which is approximately 1,000 times lower than levels of sTNFR1 (942 ± 32 ng/ml) and sTNFR2 (2,587 ± 76 ng/ml). The sTNFR2 is then a high capacity, low affinity receptor for TNF-α that has no known signal transduction activity. The sTNFR2 does not include a clearance mechanism as is observed in etanercept which is composed of both TNFR2 ligand binding domain but also an Fc domain providing for clearance. Even with the Fc domain fused to the TNFR CRD, TNFα levels are higher in psoriasis patients taking etanercept compared to adalimumab and infliximab. The second interpretation is consistent with clinical observations of poor outcomes and treatment failures associated with elevated sTNFR2.

## 5. Vaccine strategies for chronic inflammation

Vaccines for management of infectious diseases have historically used large antigens such attenuated (yellow fever, measles, mumps, and rubella), inactivated (polio, rabies, influenza, and hepatitis A) or subunit (Hepatitis B, human papillomavirus, and influenza) pathogens. The large antigens are preferred because their multiple epitopes offer a hedge against variation in pathogens and limit immune evasion. However, use of pathogens as large antigens in vaccines can be associated with significant adverse events. Reactivation of an attenuated tuberculosis vaccine (BCG) was administered to 251 neonates were given three oral doses in Lubeck Germany in 1930. 173 infants developed signs of TB and 72 died (50). A diphtheria vaccine unknowingly contaminated with *Staphylococcus aureus* was administered to 21 children in Bundaberg Australia in 1928. 12 of the children died within 2 days of vaccination (51). Large antigens may also increase the potential for autoimmune host reactions. The US FDA prohibited (21 CFR 610.19) Group A streptococcus organisms or their derivatives from vaccines because they may induce dangerous tissue reactions in humans. The action was based on administration of an M protein vaccine to 21 healthy siblings of patients with rheumatic fever. Two of the individuals developed rheumatic fever and another developed possible rheumatic fever (52). The FDA revoked the specific requirements for *S. pyogenes* vaccine in 2006 and now peptide-based vaccines utilize segments of the M-protein as well as non-M protein antigens (53). Vaccines designed to target self-protein targets do not share the need for large antigens as there is minimal opportunity for immune evasion.

Large antigens present numerous epitopes to the immune system and can result in “immune confusion.” Some surface-exposed regions of the antigen are associated with subdominant or weak antibody responses in contrast to immunodominant epitopes (54). For example, Dengue virus interacts with host cell receptors with a fusion loop (FL) of the M protein. While the FL domain is an immunodominant region, it leads to poorly neutralizing antibodies while other sites in the M protein are subdominant but elicit protective antibodies (55). Dengue vaccine development has been hampered by this situation. Another example involves the hemagglutinin (HA) of influenza A virus (IAV). The highly conserved HA stem region is associated with protective antibodies, but this site is subdominant (56). This explains why IAV vaccines need to be updated for each season's infection. Vaccines with large antigens present both immunodominant and subdominant regions and apparent immune confusion with respect to efficacy.

Current trends utilize smaller peptides to avoid the complexity of reactions to large antigens. Peptide vaccines are synthetically prepared so the antigen can be fully characterized. The production is highly reproducible, fast, and cost-effective. The vaccines are generally water soluble and may be stored without refrigeration. The small size limits potential for allergic or autoimmune responses.

Peptide-based anticancer vaccines targeting human papilloma virus (HPV) utilize 7–14 amino acid epitope sequences targeting the E5, E6, and E7 proteins of HPV-16. The linear B-cell epitopes effectively activate CD8 + cell responses but lack Th epitopes. The small peptides are not promptly recognized by antigen presenting cells (APC) so immunostimulatory adjuvants and vaccine formulations are necessary to ensure vaccine efficacy. A universal T helper epitope that covers a broad range of HLA alleles called PADRE (AKFVAAQTLKAAA) is more stimulatory that natural Th epitopes (57). Immunostimulant peptides can be added including influenza hemagglutinin CD4 + peptide (HA_307–319_; PKYVKQNTLKLAT) or tetanus toxoid peptide (TT_830–844_; CG-QYIKANSKFIGITEL) (58).

### 5.1. Epitope-peptide based vaccines

Active immunization with vaccines targeting TNF-α are being developed to address the cost and antidrug antibody (ADA) limitations of current TNF-α inhibitors including infliximab, adalimumab and etanercept. Initial attempts employed the sTNF-α molecule as the immunogen including TNF-K and TNF AutoVaccine. These approaches are effective in animal model studies (59) but were not successful in human clinical trials (60).

An epitope-based vaccine pursues design for optimal target specificity (61). The result is a vaccine with fewer allergenic and reactogenic effects (62). The key limitation is small peptides are inherently poor in immunogenicity imposing requirements for an appropriate scaffold and adjuvant. A TNF epitope vaccine, CRM197, has been described which employs the epitope peptide (AA 80–97) (63) from monoclonal antibody binding and a transmembrane domain of the diphtheria toxoid as adjuvant (64).

Alternative splicing describes how multiple transcripts can be created from a single pre-mRNA, which are then translated into a family of protein isoforms. The splicing process is controlled by spliceosomes, splice sites, and splicing elements for splicing factors. Alternative splicing is associated with many cardiac diseases including hypertrophic cardiomyopathy (alternate splicing of myomesin and troponin), myotonic dystrophy type 1 (DM1; alternate splicing of dystrophia myotonica protein kinase), Brugada syndrome (abnormal splicing of SCN5A), dilated cardiomyopathy, ischemic cardiomyopathy, and atherosclerosis (65). Alternative splicing of apoptotic pathways plays a central role in development of cardiovascular disease with alternate splice forms of TNF-α. The alternate spliced protein isoforms may represent neoantigens that may be exploited by vaccines.

Vaccination against atherosclerosis as a potential effective approach has been under investigation for more than 20 years. Different antigens have been tested in animals with a great success. Lipid-related antigens like Ox- LDL, PCSK9, non-lipid related antigens like interleukins, HSPs β2GPI, DNA vaccination and whole cell vaccination are some examples of successful examinations in animals (66). Plant-based vaccination which has some advantages over traditional methods has recently attracted the attention of the scientific community.

AtheroVax is a therapeutic vaccine for individuals with ongoing coronary artery disease. The vaccine antigen is an alternately spliced variants of the tumor necrosis factor alpha receptor 2 (TNFR2). The TNF-alpha inflammation pathway is an important driver of atherosclerosis and formation of vascular plaque. Targeting the disease associated neoantigen, sTNFR2, that exploits the novel pre-mRNA joining of exon 6 to exons 8 and/or 9 thus sparing the membrane bound, wild-type, TNFR2 (Table 4). The vaccine is expected to have a long duration of action that can replace current therapies that while effective, require chronic treatment resulting in frequent failures due to lack of patient compliance.

**Table 4:** Rationale for targeting sTNFRII.

Observation	References
TNFα has a very short half-life, 1.2–2 h	(15)
Relationship between [TNFα] and sTNFRII is not inverse	(47)
Associated with sites of inflammation, arthritis in Bechet's disease.	(41)
Associated with cardiovascular disease in diabetics	(42)
Associated with increased risks of cardiovascular events and mortality in patients with stable coronary heart disease	(46)
Psoriasis patients treated with TNFα inhibitors produce more sTNFα and sTNFR2 and patients not responding produce higher levels of both sTNFα and sTNFR2	(47)
Associated with all-cause mortality or an increased risk for cardiovascular events in advanced CKD	(45)
sTNFR2 levels were linked to epigenetic regulation associated with proinflammatory gene expression in lymphocytes from participants in the Framingham Heart Study	(43)

The sTNFR2 is a soluble extracellular protein observed in blood plasma. A vaccine producing humoral responses (antibodies) more prominently than cellular (cytotoxic T-cell) responses is desired. Aluminum-based adjuvants such as aluminum hydroxide (Alum) are poor stimulators of cellular immune responses.

## 6. Conclusions

A therapeutic vaccine for the treatment of atherosclerotic CVD targeting chronic inflammation supported by expression of the sTNFRII(Δ7) splice variant is described. The approach to the vaccine is based on the premise that chronic inflammation is a key driver of CVD. TNFα and the associated signal transduction pathway is central to the maintenance of chronic inflammation. While numerous anti-TNFα therapeutics are currently available, they have limitations for use in treating CVD including variable responses and emergence of antidrug antibodies (ADA). An understanding of the diverse forms of the TNF receptors may explain the variable responses to TNFα ultimately focusing attention to the TNFRII. The TNFRII receptor is further complicated by the expression of both membrane bound and at least two soluble forms. The proteolytically cleaved soluble form sTNFRII(PC) is distinct in structure and function from the alternate splice form sTNFRII(Δ7). The small peptide vaccine, AtheroVax, is a linear B-cell epitope created by joining exons 6–8 which is unique to the sTNFRII(Δ7) and is not expected to cross react with the membrane bound (mTNFRII) or sTNFRII(PC). The resulting therapeutic vaccine specificity supports chronic use in limiting the development and progression of atherosclerosis.

AtheroVax is a unique vaccine for atherosclerotic CVD by targeting chronic inflammation and not the process of cholesterol synthesis, transport, and states of oxidation. The proposed benefit is summarized in a target product profile (Table 5). The treatment regimen is expected to be once to twice a year which is expected to limit cost and support compliance to the treatment regimen. The expectation of low cast and anticipated product stability may even support acceptance as a global therapy for CVD.

**Table 5:** Target product profile (TPP).

Product properties	Minimum acceptable	Ideal
Primary indication	Prevention of atherosclerosis progression	Prevention of atherosclerosis
Patient population	Adults with atherosclerosis who are at risk for progression to MACE	Adults at risk for atherosclerosis
Treatment duration	Chronic	Chronic
Delivery mode	Injection (IM, SC, ID)	Subcutaneous injection
Dosage form	Sterile liquid with adjuvant	Sterile dry powder for rehydration in isotonic saline
Regimen	Once/6 months	Once/year
Efficacy	Equal to current SOC	Better outcomes than SOC
Risks/side effects		Local injection site reactions treated with NSAIDs

The potential successful development of AtheroVax is likely to be used in conjunction with other treatments such as standard of care medications and lifestyle modifications.
